# Cognition-Modulated EEG Signatures and Clinical Features in Major Depressive Disorder: A Machine Learning-Based Exploratory Study

**DOI:** 10.31083/AP50791

**Published:** 2026-06-28

**Authors:** Cheng-Ta Li, Chih-An Lai, Jia-Shyun Jeng, Chih-Ming Cheng, Ya-Mei Bai, Chung-Ping Chen

**Affiliations:** ^1^Precision Depression Intervention Center (PreDIC), Department of Psychiatry, Taipei Veterans General Hospital, 112 Taipei, Taiwan; ^2^Department of Psychiatry, Faculty of Medicine, National Yang Ming Chiao Tung University, 112 Taipei, Taiwan; ^3^Institute of Brain Science and Brain Research Center, National Yang Ming Chiao Tung University, 112 Taipei, Taiwan; ^4^Graduate Institute of Biomedical Electronics and Bioinformatics, College of Electrical Engineering and Computer Science, National Taiwan University, 106319 Taipei, Taiwan

**Keywords:** major depressive disorder, electroencephalography, machine learning, treatment-resistant depression, suicidal ideation

## Abstract

**Background:**

Major depressive disorder (MDD) includes heterogeneous clinical dimensions, including depressive symptom severity, treatment refractoriness, and suicidality, which are commonly assessed using subjective rating scales and retrospective clinical histories. Dysfunction of frontal and anterior cingulate cortex (ACC) networks has been implicated in MDD, suggesting that electroencephalography (EEG)-based approaches combined with machine learning (ML) may help with objective characterization of clinical heterogeneity.

**Methods:**

Resting-state and cognition-modulated EEG data were analyzed from 209 patients with MDD. A rostral ACC-engaging cognitive task (RECT) was used to probe frontal-ACC circuitry. Linear and non-linear EEG features extracted from frontal electrodes across multiple frequency bands were integrated with several ML classifiers to perform exploratory classification of suicidality, depressive symptom severity, and treatment refractoriness. Class imbalance in suicidality was addressed using synthetic oversampling applied to the training data only.

**Results:**

ML models, particularly Random Forest (RF), outperformed support vector machines across all outcomes. RF achieved classification accuracies of around 83% (area under curve (AUC) = 0.83) for depression severity and 87% (AUC = 0.87) for treatment refractoriness. Suicidality categorization performance improved following data balancing. Feature importance studies found consistent patterns across outcomes, with useful predictors primarily obtained from frontal electrodes and nonlinear EEG complexity measures.

**Conclusions:**

The combination of cognition-engaging frontal modulation and ensemble-based ML applied to EEG data suggests the feasibility of an exploratory, unified EEG-based approach for identifying important characteristics of MDD. These findings highlight the importance of frontal network dysfunction across the severity spectrum of MDD and the need for further validation in larger and longitudinal cohorts.

## Main Points

1. Electroencephalography (EEG) and machine learning were used to classify depression severity, treatment refractoriness, and suicidality in patients with major depressive disorder (MDD).

2. Random Forest showed the best performance, achieving 83% accuracy for depression severity and 87% accuracy for treatment refractoriness.

3. Frontal EEG signals and nonlinear complexity measures were the most important predictive features across outcomes.

4. These findings support frontal-cingulate dysfunction as a key biomarker of MDD heterogeneity and highlight the potential of EEG-based precision psychiatry.

## 1. Introduction

Major depressive disorder (MDD) is expected to become the primary cause of disability among people in 2030, according to estimates from the World Health Organization [1]. Without adequate treatment, depression imposes substantial individual and societal burdens, but a significant proportion of patients with MDD do not respond despite several medication treatments as shown by the famous Sequenced Treatment Alternatives to Relieve Depression (STAR*D) study [2]. Such treatment-resistant depression (TRD) is linked to poor clinical and psychosocial outcomes [3]. In fact, MDD is a highly heterogeneous condition with substantial variability in symptom presentation and treatment response. Recent neuroimaging studies suggest that this heterogeneity may reflect divergent patterns of large-scale neural network and information processing [4]. Furthermore, more than 700,000 individuals worldwide die by suicide, and people with depression face a markedly elevated risk compared with the general population [5]. TRD patients have a significantly higher risk of suicide than non-TRD patients [6].

Frontal abnormalities, particularly involving the prefrontal cortex (PFC), play a central role in the pathophysiology of major depressive disorder [7,8]. TRD is associated with PFC hypometabolism [9] and fronto-limbic dysregulation [10], and neuromodulation targeting the left PFC with repetitive transcranial magnetic stimulation (rTMS) [11] or intermittent theta-burst stimulation (iTBS) [12,13,14] has demonstrated antidepressant efficacy in patients with inadequate medication response. In addition, ketamine, an N-methyl-D-aspartate (NMDA) antagonist, has been linked to the glutamatergic pathophysiology of depression [15]. Low-dose ketamine has fast antidepressant effects in patients with TRD, possibly due to modulation of frontal and fronto-limbic dysfunction [16].

Growing evidence links electroencephalography (EEG) measures to frontal and anterior cingulate cortex activity and antidepressant treatment response in MDD [17,18]. In particular, frontal theta activity has been associated with treatment outcomes [18]. Using combined EEG and fluorodeoxyglucose-positron emission tomography (FDG-PET), we previously demonstrated that rostral anterior cingulate cortex (rACC)-engaging cognitive task (RECT)-induced increases in frontal theta power were correlated with rostral ACC metabolism and were associated with response to rTMS [19]. However, findings based on resting frontal theta have been inconsistent, highlighting the need for more sensitive and task-informed approaches [20].

Recent meta-analyses and practical investigations have shown that EEG-based machine learning (ML) models can consistently predict how people with MDD would respond to antidepressants. The pooled classification accuracy is about 84% and the area undercurve (AUC) is about 0.85 [21]. Furthermore, recent work applying ensembles and complex EEG feature extraction has successfully predicted response to neuromodulation treatments such as rTMS [22]. In a large, combined cohort of patients receiving 10-Hz rTMS or iTBS over the left PFC, we found that pre-treatment cognition-modulated frontal EEG features could accurately predict antidepressant response [22]. By combining linear (e.g., frontal theta) and nonlinear EEG features that show signal trend, stability, and complexity, ML systems were able to make far more accurate predictions than traditional methods. For example, Random Forest (RF) achieved accuracies of 83.3% for rTMS and 88.9% for iTBS, surpassing traditional support vector machines and logistic regression models that relied simply on frontal theta.

Suicidality is among the most severe and clinically significant aspects of MDD and constitutes a primary cause of premature mortality. Significantly, converging neuroimaging and neurophysiological data indicate that suicide ideation and behavior are linked to abnormalities in frontal-cingulate networks, specifically implicating the PFC and ACC [17]. These brain regions are also very important for controlling emotions and thoughts, which are often abnormal in MDD. Consequently, suicidality might be regarded as a clinically significant aspect of MDD that may share common neurobiological causes with the intensity of depressive symptoms and treatment resistance.

In parallel, MDD has a wide range of clinical severities, with depressed symptom burden and treatment refractoriness being two independent but linked characteristics. While symptom severity indicates the current clinical condition, treatment refractoriness describes the inability to obtain adequate recovery despite suitable therapies. Importantly, TRD is now viewed as a continuum rather than a binary categorization, which complicates therapeutic care and research comparability [23].

With recent breakthroughs in artificial intelligence, ML techniques can objectively identify informative EEG patterns and create clinically significant predictions in MDD. More evidence suggests that electrophysiological measures derived from EEG may reflect neural network dynamics underlying depressive illness, such as cortical rhythmic activity and nonlinear signal complexity. Building on evidence that frontal and ACC dysfunction play a central role in depressive severity, treatment refractoriness, and suicidality, the current study investigated whether combining a cognition-engaging modulation paradigm (i.e., RECT) [19], which selectively engages frontal-ACC activity, with modern machine learning algorithms could improve the characterization of these key clinical dimensions of MDD [22]. Specifically, we combined linear spectral characteristics and nonlinear complexity measures collected from resting-state and cognition-modulated EEG recordings with several machine learning models to investigate prediction frameworks for these clinically significant dimensions.

## 2. Materials and Methods

### 2.1 Study Overview and Clinical Assessments

In this retrospective study, we collected EEG data from patients with MDD. Eligible patients were adult patients aged from 21 to 70 years and diagnosed with recurrent MDD on the basis of Diagnostic and Statistical Manual of Mental Disorders, Fifth Edition (DSM-5) criteria. Diagnoses were mainly established after taking a thorough medical history and conducting a semistructured interview by administering the Mini International Neuropsychiatric Interview (MINI). The recruited patients were required to be antidepressant-free for at least 1 week before EEG experiments and clinical assessments. Hamilton Depression Rating Scale (HDRS-17) was used for evaluating severity of depressive symptoms [24]. The degree of treatment refractoriness was measured using the Maudsley Staging Method (MSM) [25]. The MSM is a point-based staging model and incorporates three main factors to cover many dimensions of treatment resistance: treatment (i.e., numbers of antidepressant treatment failures and whether augmentation or electroconvulsive therapy had been used), severity of symptoms, and duration of presenting episode. Patients were included if they met recruitment criteria between May 1, 2018 and Feb 12, 2023. Participants who had been diagnosed with other major psychiatric disorders, such as schizophrenia, psychotic disorders, or bipolar disorder, were excluded. Individuals with neurological illnesses that may impair brain function or EEG signals, such as stroke, epilepsy, neurocognitive problems, or brain tumors, were also excluded. Other exclusion criteria included a history of brain surgery and the presence of intracranial implants (e.g., neurostimulators). 

### 2.2 EEG Acquisition and RECT Modulation

Cognition-modulated EEG (RECT) was acquired in a controlled, electrically shielded environment with participants seated and eyes closed [19]. EEG was recorded using a standard 32-channel cap (10–20 system; Ag/AgCl electrodes; impedance <5 kΩ) with linked-mastoid reference and FP1-FP2 ground, , using NuAmps amplifier (Compumedics Neuroscan, Charlotte, NC, USA) and Neuroscan software (version 4.3; Compumedics Neuroscan). Signals were sampled at 1000 Hz and band-pass filtered at 1–60 Hz, and vertical and horizontal eye movements were monitored using electrooculogram electrodes (Compumedics Neuroscan, Charlotte, NC, USA) [19].

The RECT program was primarily based on the flexibility task of the Tests for Attentional Performance [26] and had been found to induce and modulate brain activities in the rACC [19]. During the task, competing stimuli (sharp and round forms) were presented simultaneously on the left and right sides of the computer screen, and the patients were asked to press a response key indicating the correct side of the screen containing the target stimuli with their index finger. To ensure complete participation in the program, patients completed 10-minute continuous testing trials under the guidance of a qualified research assistant following the pretest trials. As long as the patient paid close attention for the whole ten minutes of the RECT program, no minimum accuracy was needed. The shape task, instead of a color task, was used for the target stimuli of the RECT program because the former had been found to be associated with more frontal theta engagement than the latter [27]. Resting-state five-minute EEG datasets were acquired before and right after performing RECT (i.e., baseline RECT and post-RECT).

### 2.3 EEG Data Preprocessing

To remove unwanted signals such as eye blinks and movements, muscle artifacts, cardiac signals, and random noises, raw EEG data were filtered by a band-pass finite-duration impulse response (FIR) filter with Hamming window and then Independent Component Analysis (ICA) [28] was applied (details of the ICA method, please refer to the **Supplementary Material** online). In brief, artifact components were identified based on a combination of spatial topography, spectral characteristics, and time-course patterns. For example, ocular artifacts were identified by specific frontal scalp distributions and massive slow deflections that corresponded to eye blinks or movements. In addition, muscle artifacts were identified by high-frequency activity with localized temporal or peripheral scalp distributions and cardiac artifacts were identified by rhythmic activity synchronized with the cardiac cycle. Components showing these characteristic features were removed prior to EEG feature extraction. After ICA, artifact-free EEG data from seven frontal channels (FP1, FP2, F7, F3, Fz, F4, F8) were retained for analysis and were divided into five frequency bands: delta (1–4 Hz), theta (4–8 Hz), alpha (8–13 Hz), beta (13–30 Hz), and gamma (30–60 Hz). EEG data in the frontal region (FP1, F7, F3, Fz, F4, F8, and FP2) were of our main interest, because frontal function plays an important role in the pathophysiology of MDD [9,29] and it could cover frontal theta activity that we reported to have a value in predicting responses to rTMS over PFC [19]. These channels (FP1, FP2, F3, F4, F7, F8, and Fz) were chosen to study neural dynamics in the PFC and ACC networks, which are strongly implicated in MDD and directly engaged by the RECT paradigm [19]. Therefore, limiting analysis to frontal channels allowed us to concentrate on the most theoretically important cortical regions while reducing feature dimensionality and enhancing model stability.

### 2.4 Feature Extraction

From each channel, we computed six linear and nonlinear features as described below. Non-linear features of EEG were extracted by Largest Lyapunov Exponent (LLE) [30], Detrended Fluctuation Analysis (DFA) [31], Approximate Entropy (ApEn) [32], Katz Fractal Dimension (KFD) [33], and Higuchi Fractal Dimension (HFD) [34]. The Welch method was applied to extract linear features by computing the band power of EEG signals [35]. For detailed information, please see the online **Supplementary Material **and **Supplementary Fig. 1**.

### 2.5 Feature Selection, Classification, and Machine Learning Methods

Machine learning analyses were implemented using Python with the scikit-learn library. Four classification algorithms were evaluated: support vector machine (SVM),RF, XGBoost, and CatBoost (details please refer to the **Supplementary Material** online and our previous publication [22]). For each analysis, the dataset was randomly divided into training and testing subsets using an 80:20 split [36]. This procedure was repeated ten times to reduce sampling variability. Stratified sampling was applied to preserve the class distribution across training and testing datasets. Hyperparameters for each model were optimized using grid search within the training dataset. Model performance was evaluated on the independent test dataset using accuracy, area under the receiver operating characteristic curve, precision, recall, and F1-score. For the best classification and regression tree based bagging and boosting algorithm, the top five most important features would also be investigated by calculating values of feature importance using scikit-learn (version 1.2.1; scikit-learn developers, Paris, France) running under a Python 3.11 environment (higher values, more important feature) for exploratory purposes. Feature importance estimation and model training were conducted exclusively within the training dataset, while the test dataset remained completely independent for final performance evaluation.

Given the imbalance in suicide severity categories and fewer samples representing high-risk people, we used the Synthetic Minority Oversampling Technique (SMOTE) [37] to improve model training. SMOTE artificially augments minority classes by interpolating between surrounding samples, minimizing bias toward less severe majority classes. SMOTE was implemented using the conventional approach with k = 5 nearest neighbors and was limited to the training dataset to prevent information leakage. To ensure the integrity of performance evaluation, oversampling was performed solely to the training set, leaving the validation and test sets unchanged.

In contrast, for analyses of depression severity and treatment refractoriness, age- and sex-matched subsamples from the original cohort were established and synthetic oversampling was not used. Participants were matched between comparison groups to ensure similar age and gender distributions while preserving a balanced sample size. The matched datasets were exclusively used for the classification analysis in this exploratory research.

### 2.6 Outcome Measures

Standardized clinical assessments were used to determine suicide severity, depression severity, and therapy refractoriness. Suicide severity was measured using item 3 of the HDRS-17, which rates suicidal ideation and related behaviors on an ordinal scale. Suicidality was divided into five stages based on the conventional HDRS score (0–4). In this study, HDRS item 3 served as a pragmatic proxy for the severity of suicidal ideation. Depression severity was assessed based on baseline HDRS-17 total scores. Low severity was designated as HDRS-17 <17 and high severity as HDRS-17 ≥17. This cutoff was chosen to distinguish mild from at least moderate depressive severity, consistent with recommended HDRS-17 severity ranges in prior studies [38,39]. Treatment refractoriness was indexed using the antidepressant treatment failure subscore of the MSM [25]. Low refractoriness was defined as a score of 0–1, whereas high refractoriness was defined as a score ≥2. This operational cutoff was selected to distinguish patients with fewer than two antidepressant treatment failures from those with at least two failed trials, consistent with the dimensional framework of the MSM and with the commonly used clinical concept of treatment-resistant depression.

### 2.7 Statistical Analysis

Because the present study aimed to develop exploratory ML models rather than to test a predefined statistical hypothesis, a formal a priori sample size calculation was not performed. Instead, the sample size was determined by the number of eligible patients available in our clinical EEG database during the study period. The final dataset included 209 patients with MDD, which is larger than or comparable to sample sizes reported in many EEG-based machine learning studies of depression [40,41]. To reduce the risk of overfitting when analyzing high-dimensional EEG features, model training and evaluation were conducted using repeated train-test splits and cross-validation procedures.

One-way analysis of variance (ANOVA) and independent *t*-test were used to compare continuous variables (e.g., age, HDRS-17) across three groups and two groups, respectively. Fisher’s exact test and/or Yates’s correction were used to compare categorical variables (e.g., responders) between groups. To evaluate whether model performance exceeded chance-level classification, we assessed accuracy relative to the null-information rate (NIR), defined as the largest class proportion, using a one-tailed binomial test [42,43]. With *n* being the amount of the whole data, *x* being the sum of true positive samples in each class, and *p* being NIR, the *p*-value (ACC>NIR) is calculated as


$$p-value_{\left(ACC>NIR\right)}=1-\sum_{k=1}^{x}\left(\frac{n}{k}\right)p^{k}(1-p{)}^{n-k}$$

Except for EEG data, all statistical analyses were performed using SPSS 21.0 (IBM, Armonk, NY, USA). Statistical significance was set at *p* < 0.05 (two-sided tests).

## 3. Results

A total of 209 MDD patients were included in the analysis, comprising 85 males and 124 females. The mean age of the sample was 38.6 ± 14.2 years. The mean and standard deviation (SD) of baseline depression severity, as measured by HDRS-17, was 21.8 ± 6.6. The mean (SD) of MSM score was 8.6 ± 2.0, reflecting substantial variability in treatment refractoriness across participants. Of the 209 patients, 17.7% (n = 37) had no suicidality, 23.0% (n = 48) had mild, 43.1% (n = 90) had moderate, 13.4% (n = 28) had severe, and 2.9% (n = 6) had profound suicidality (**Supplementary Table 1**).

### 3.1 Suicide Severity Classification

Table 1 summarizes the model performance for suicide severity classification. When trained on the original imbalanced dataset with five ordinal classes (0–4), all classifiers performed poorly (left panel; Table 1). Overall accuracy ranged from 19.0% to 30.9%, with only the CatBoost model performing significantly better than the no-information rate (ACC>NIR, *p* = 0.030), demonstrating that multi-class suicide severity categorization is especially difficult under substantial class imbalance.

**Table 1.** **Comparison of machine learning model performance in suicide severity classification before and after applying Synthetic Minority Oversampling Technique (SMOTE)**.

	Before SMOTE				After SMOTE			
Classification	SVM	Random Forest	XGBoost	CatBoost	SVM	Random Forest	XGBoost	CatBoost
Accuracy	0.190	0.286	0.262	0.310	0.476	0.976	0.595	0.524
AUC	0.578	0.480	0.473	0.471	0.540	0.999	0.637	0.719
Precision	0.184	0.179	0.168	0.180	0.458	0.977	0.626	0.478
Recall/Sensitivity	0.190	0.286	0.262	0.310	0.476	0.976	0.595	0.524
Specificity	0.800	0.824	0.786	0.833	0.758	0.970	0.829	0.788
F1-score	0.176	0.217	0.204	0.204	0.462	0.976	0.566	0.494
*p*-value (ACC>NIR)	0.469	0.062	0.118	0.030*	<0.001*	<0.001*	<0.001*	<0.001*

*p < 0.05. AUC, area under curve; ACC, accuracy; NIR, null-information rate; SVM, support vector machine.

To mitigate this imbalance, the SMOTE was applied to the training data only, while the test dataset remained unchanged. After balancing class distributions (right panel; Table 1), classification performance improved across models. The RF classifier showed the highest apparent performance (accuracy = 97.6%), whereas other models also exceeded chance-level classification (all *p* < 0.001). However, given the small number of high-risk cases and the use of synthetic oversampling, these results should be interpreted cautiously and primarily as exploratory, reflecting improved model learnability rather than definitive classification performance.

### 3.2 Depression Severity Classification

After age- and sex-matching, the analytic subsamples comprised 72 participants in each depression severity group and 75 participants in each treatment refractoriness group. Table 2 shows the model’s performance in classifying depression severity. Among all the evaluated classifiers, the RF model had the best overall performance, with an accuracy of 82.8%, an AUC of 0.83, and an F1-score of 0.83, which exceeded the no-information rate (*p* = 0.0004). Ensemble-based approaches such as RF, XGBoost, and CatBoost outperformed SVMs in terms of stability. In comparison, the SVM classifier did not perform substantially better than chance (ACC>NIR, *p* = 0.607), indicating that it is only moderately efficient for predicting depression severity in this dataset.

**Table 2.** **Classification results of different machine learning models for depression severity**.

Classification	SVM	Random Forest	XGBoost	CatBoost
Accuracy	0.655	0.828	0.724	0.759
AUC	0.605	0.829	0.750	0.774
Precision	0.625	0.800	1.000	0.667
Recall/Sensitivity	0.714	0.857	0.500	0.923
Specificity	0.600	0.800	1.000	0.625
F1-score	0.667	0.828	0.667	0.774
*p*-value (ACC>NIR)	0.607	<0.001*	0.003*	0.002*

*p < 0.05.

### 3.3 Treatment Refractoriness Classification

The classification results for treatment refractoriness are given in Table 3. Ensemble-based approaches outperformed SVMs in terms of performance stability, which was consistent with the findings for depression severity. The RF model outperformed the no-information rate, with an accuracy of 86.7%, an AUC of 0.87, and an F1-score of 0.86 (*p* < 0.001).

**Table 3.** **Classification results of different machine learning models for treatment refractoriness**.

Classification	SVM	Random Forest	XGBoost	CatBoost
Accuracy	0.733	0.867	0.733	0.800
AUC	0.813	0.867	0.741	0.819
Precision	0.684	0.923	0.833	0.929
Recall/Sensitivity	0.867	0.800	0.625	0.722
Specificity	0.600	0.933	0.857	0.917
F1-score	0.765	0.857	0.714	0.813
*p*-value (ACC>NIR)	0.305	<0.001	0.007*	0.001*

*p < 0.05.

While XGBoost and CatBoost performed much better than chance, the SVM did not attain accuracy above the no-information rate (ACC>NIR, *p* = 0.31), despite its relatively high sensitivity. These findings suggest that non-linear ensemble models may be more effective than SVM in capturing EEG patterns related with antidepressant treatment refractoriness in this dataset.

### 3.4 Feature Importance Analysis: Suicide Severity

Given the SVM’s unstable performance, feature significance studies were limited to the RF, XGBoost, and CatBoost models. Across various ensemble techniques, the most frequently selected features for suicide severity classification (Table 4) were mainly obtained from frontal electrodes (Fz, FP1, and F7). From a spectral perspective, gamma-band features were most prominent, followed by delta and alpha bands. Features that appeared among the top-ranked predictors in at least two ensemble models were considered convergent features. The convergence of feature selection across ensemble models suggests that frontal gamma-band complexity features may be relevant to exploratory characterization of suicide severity.

**Table 4.** **Key EEG features identified across ensemble models for suicide severity classification**.

Electrode	Feature	Sub-band	RF	XGBoost	CatBoost
Fz	HFD	Gamma	✓	–	✓
FP1	KFD	Gamma	✓	–	✓
FP1	HFD	Gamma	–	✓	✓
F7	HFD	Delta	✓	–	✓
F3	HFD	Beta	–	✓	–
FP2	KFD	Alpha	✓	–	–

RF, Random Forest.

### 3.5 Feature Importance Analysis: Depression Severity

For depression severity classification (Table 5), feature importance patterns showed a preference for frontal electrode sites, specifically FP2, F7, FP1, and F8, with FP2 and F7 emerging as the most frequently selected channels across all three models. Linear and non-linear features contributed relatively equally, with alpha-band features being the most typically selected, followed by delta, gamma, and theta bands.

**Table 5.** **Key EEG features identified across ensemble models for depression severity classification**.

Electrode	Feature	Sub-band	RF	XGBoost	CatBoost
FP2	DFA/ApEn/KFD	Alpha/Beta	✓	✓	✓
F7	HFD/ApEn/LLE/Welch	Delta/Gamma/Alpha	✓	✓	✓
FP1	DFA/KFD	Alpha/Theta	✓	–	✓
F8	LLE/Welch	Delta/Theta	–	✓	✓
F4	HFD	Gamma	–	✓	–
F3	Welch	Alpha	✓	–	–

ApEn, Approximate Entropy; LLE, Largest Lyapunov Exponent.

Only a small number of features showed statistically significant group differences, implying that depression severity classification remained difficult at the individual feature level, despite good model-level performance.

### 3.6 Feature Importance Analysis: Treatment Refractoriness

Feature importance studies for treatment refractoriness classification (Table 6) revealed that features coming from frontal electrodes, specifically FP2, FP1, and F4, were the most commonly selected across ensemble models, with FP2 having the most informative features. Spectrally, theta-band characteristics predominated, followed by delta, beta, and gamma bands. Linear and non-linear characteristics were chosen at similar rates, while LLE-based features were less consistently represented. These findings suggested that treatment refractoriness cannot be easily identified using single EEG parameters alone, but may instead be reflected in multivariate patterns collected by ensemble-based approaches.

**Table 6.** **Key EEG features identified across ensemble models for treatment refractoriness classification**.

Electrode	Feature	Sub-band	RF	XGBoost	CatBoost
FP2	HFD/DFA/KFD	Gamma/Theta/Beta	✓	✓	✓
FP1	Welch/DFA	Theta/Alpha	✓	✓	–
F4	ApEn	Delta	–	✓	✓
F3	KFD/DFA	Theta	✓	–	–
F7	Welch	Beta	–	✓	–
F8	LLE/KFD	Delta/Theta	–	–	✓

## 4. Discussion

The present study examined whether combining a cognition-engaging modulation paradigm (RECT), which selectively engages frontal and ACC activity, with modern ML approaches can facilitate the exploratory characterization of major clinical characteristics of MDD. By integrating linear and non-linear EEG features derived from recordings obtained before and after RECT with multiple classification algorithms, we evaluated EEG-based patterns associated with suicidality, depressive symptom severity, and treatment refractoriness within a unified analytic framework. These findings are also consistent with recent neuroimaging studies showing that mood disorders are heterogeneous and associated with large-scale reorganization of cortical functional architecture [4,44].

### 4.1 Overall Model Performance and Clinical Implications

Across all three clinical dimensions, ensemble-based ML models, particularly RF, consistently outperformed SVM. This pattern was consistent with our prior work in rTMS-related EEG studies [22], implying that ensemble approaches may be more suitable for capturing complex EEG patterns associated with frontal network modulation and treatment-related neuroplasticity in MDD. This advantage may partly reflect the heterogeneous nature of the EEG feature set, which includes both linear spectral features and nonlinear complexity measures across multiple frequency bands, resulting in nonlinear interactions that tree-based ensemble models are particularly well suited to capture. This advantage was evident for depression severity and treatment refractoriness classification, and for suicide severity after addressing class imbalance. Together with prior evidence linking EEG measures to depressive symptom severity [45], these findings support the use of non-linear, tree-based ensemble models for multivariate EEG characterization. Importantly, the present study did not rely on a single machine learning model. Instead, several algorithms were systematically compared to provide a more objective evaluation of EEG-derived features. The observation that ensemble-based models outperformed the SVM classifier suggests that the detected patterns are not dependent on a single modeling strategy.

From a clinical perspective, these results indicate that EEG-based approaches may complement conventional symptom-based and historical assessments when characterizing depression severity and treatment refractoriness. Such objective electrophysiological information may be particularly useful in situations where symptom reporting fluctuates or when early identification of patients at risk for severe illness or treatment resistance is clinically important. However, given the exploratory and cross-sectional nature of the study, the present findings should be interpreted as supporting feasibility, and further validation in independent and longitudinal cohorts is required before clinical application [46].

### 4.2 Suicide Severity Classification and the Role of Data Imbalance

Suicide severity classification was much more challenging than the other outcomes, especially in the presence of class imbalance, which is a well-known issue in ML-based suicide risk estimation [47]. Under these conditions, all classifiers performed poorly, highlighting the intrinsic difficulties of multi-class suicidality prediction and the significant disparity among severity levels. Model performance improved significantly after SMOTE was applied exclusively to training data.

The marked improvement in RF performance after SMOTE should be interpreted cautiously. Synthetic oversampling can overstate class separability in high-dimensional, imbalanced datasets, resulting in overly optimistic performance estimates, especially in multi-class scenarios with few samples [48]. As a result, the main importance of these findings is not in absolute performance measures, but in indicating the presence of discriminatory EEG patterns associated with suicidality when higher-risk groups are sufficiently represented. Improvements across models after SMOTE further indicate that such patterns may exist but are difficult to detect without sufficient representation of high-risk individuals. The present findings should therefore be interpreted primarily as evidence of distinguishable neural signal patterns, rather than the ability to perfectly predict suicide risk.

### 4.3 Convergent EEG Feature Patterns Across Clinical Aspects

Despite variation at the single-feature level, feature importance analyses found similar patterns across ensemble models and clinical outcomes. Informative features for suicide severity, depression severity, and treatment refractoriness were primarily derived from frontal electrode sites, consistent with the involvement of frontal and anterior cingulate networks in emotion regulation and cognitive control (processes directly engaged by the RECT paradigm) [49] as well as prior findings in depression [17]. This frontal preponderance is also consistent with the neurobiological objectives of major treatment techniques for severe and resistant depression, such as rTMS and low-dose ketamine [12,14].

These findings point to convergent frontal EEG feature patterns across ensemble models. These measurements, including HFD, KFD, DFA, and ApEn, address many elements of neuronal signal dynamics, such as signal complexity, temporal correlations, and dynamical stability. Alterations in EEG signal complexity have been proposed to reflect alterations in large-scale brain network architecture and information processing in neuropsychiatric illnesses [50,51]. Importantly, ML models reveal multivariate interactions between EEG characteristics that conventional univariate statistical studies may miss.

Spectrally, gamma-, alpha-, and theta-band characteristics were prominent in suicidality, depression severity, and treatment refractoriness, respectively, indicating somewhat overlapping but different neurophysiological signatures across clinical characteristics. Gamma oscillations are thought to reflect local cortical synchronization and excitation-inhibition balance [52]. Importantly, our previous study found that gamma oscillations play an important role in severe depression, implying that gamma oscillatory dynamics could be used as electrophysiological indicators of abnormal brain network activity [53]. These findings provide support to the idea that gamma-band EEG features identified in the present ML study may reflect underlying brain mechanisms associated with emotional dysregulation and suicidality.

In contrast, theta-band features were more frequently linked to treatment refractoriness. Frontal theta activity has been consistently associated with medial prefrontal and ACC activity, and it has been proposed as a biomarker of antidepressant treatment response [17]. Together, our findings indicate that the EEG features revealed in the present study may reflect altered dynamics of fronto-cingulate networks implicated in emotion regulation, cognitive control, and treatment response in MDD.

### 4.4 Integration With RECT and a Unified EEG-Based Framework

Importantly, the convergence of frontal, complexity-based EEG features across outcomes lends support to the conceptual reason for merging RECT with EEG-based ML. RECT was developed to engage frontal and ACC circuits through cognitive regulation, and our findings suggest EEG features sensitive to these networks carry clinically important information throughout the severity spectrum of MDD. Rather than focusing on single biomarkers, the present study takes a pattern-based, multivariate approach that is more consistent with the distributed character of depressed neurobiology.

### 4.5 Limitations and Future Directions

Several limitations warrant consideration. First, although model-level performance was robust for depression severity and treatment refractoriness, individual EEG features rarely reached statistical significance, reflecting the heterogeneity and complexity of MDD. In addition, since tree-based machine learning models could capture complex multivariate interactions among predictors, individual EEG features may not necessarily demonstrate statistically significant univariate group differences. Second, suicidality classification was based on a single HDRS item, resulting in an ordinal proxy for suicidal thoughts rather than a thorough assessment of suicidal behavior or intent. Although HDRS item 3 does not encompass the entire multidimensional construct of suicide risk, it has been widely employed in depression research as an indicator of suicidal ideation severity. Future studies with specific suicide assessment instruments are required to validate these findings. Third, evaluations for depression severity and treatment refractoriness were performed on balanced subsamples rather than the entire cohort, which enhances model stability but limits generalizability to unselected clinical groups. Fourth, the cross-sectional and exploratory approaches limits drawing causal conclusions about whether the detected EEG patterns are trait markers or state-dependent effects. Finally, while a cognition-engaging task (RECT) can improve sensitivity to frontal-cingulate circuitry, it might limit the generalizability of findings to resting-state or other task contexts. Furthermore, while the overall sample size was comparable to previous EEG ML studies, several subgroups had relatively few participants. Larger, longitudinal, and externally evaluated investigations, preferably with multimodal data, are required to corroborate these findings.

## 5. Conclusions

This study demonstrated that integrating a cognition-engaging frontal modulation paradigm (RECT) with ensemble-based ML applied to EEG features can be helpful in the exploratory characterization of suicidality, depressive symptom severity, and treatment refractoriness in MDD. Ensemble models, particularly RF, showed more stable performance than conventional classifiers, although suicide severity classification remained challenging under severe class imbalance despite improvement after data balancing. Across outcomes, convergent frontal EEG complexity features were identified, consistent with established models of frontal and ACC dysfunction and with the neurobiological targets of treatments for severe and treatment-resistant depression. Together, these findings suggest the feasibility of a unified EEG-based framework for objective characterization of clinical dimensions across the severity spectrum of MDD.

## Data Availability

The data that support the findings of this study are available from Taipei Veterans General Hospital, but restrictions apply to the availability of these data, which were used under license for the current study, and so are not publicly available.
