# Supplementary material for: Cognition-Modulated EEG Signatures and Clinical Features in Major Depressive Disorder: A Machine Learning-Based Exploratory Study
Source: Alpha Psychiatry. 2026 Jun 28;27(3):50791. doi: 10.31083/AP50791 (PMC13339876; doi:10.31083/AP50791)
Supplement: Supplementary file 1 [file 2757-8038-27-3-50791-s1.zip › Supplementary Material.docx]

**Independent component analysis (ICA) and artifact removal**

Finding a linear representation of non-Gaussian data so that the components are statistically independent, or as independent as possible, is the aim of independent component analysis (ICA) [1]. The linear format of EEG signals makes it simple to eliminate artifacts like eye movement and blinking. Below is a description of the ICA algorithm [1]. Let's say we see n linear mixtures of n independent components, *x_1_, x_2_,..., x_n_*, such as $x_{j}= a_{j1}s_{1}+ a_{j2}s_{2}+\ldots+ a_{jn}s_{n}, \forall j$

Every mixture (x_j) and independent component (s_k) is assumed to be a random variable. We can assume that the independent components and the mixture variables have zero means without sacrificing generality. Let x be the random vector whose elements are the mixtures x_1, x_2,..., x_n. Similarly, let s be the random vector with elements s_1, s_2,..., s_n. Let A be the matrix with elements a_ij. The above mixing model is therefore expressed as *x=As* using this vector-matrix notation.

The equation can also be expressed as $x= \sum_{i=1}^{n} a_{i}s_{i}$, which represents the columns of matrix A. As a generative model, the ICA model explains how the components $s_{i}$ are mixed to produce the observed data. Since the independent components cannot be directly observed, they are latent variables. Furthermore, it is believed that the mixing matrix is unknown. The random vector x is all we see, and we have to use it to estimate both A and s. This needs to be carried out under the broadest possible assumptions. After estimating the matrix A, we can compute its inversive matrix A^(-1) and obtain the independent components s_1,s_2,… ,s_n by $s=A^{-1}x$.

In addition, artifact components were identified based on a combination of spatial topography, spectral characteristics, and time-course patterns. Ocular artifacts were recognized by characteristic frontal scalp distributions and large slow deflections associated with eye blinks or eye movements. Muscle artifacts were identified by high-frequency activity with localized temporal or peripheral scalp distributions, whereas cardiac artifacts were identified by rhythmic activity synchronized with the cardiac cycle. Components exhibiting these characteristic patterns were removed prior to EEG feature extraction.

**Feature extractions**

From each frontal channel (FP1, FP2, F3, F4, F7, F8, and FZ), six EEG features were extracted, including five nonlinear measures and one linear spectral feature. Nonlinear measures included the Largest Lyapunov Exponent (LLE), Detrended Fluctuation Analysis (DFA), Approximate Entropy (ApEn), Katz Fractal Dimension (KFD), and Higuchi Fractal Dimension (HFD). These features characterize different aspects of neural dynamics, including signal instability, long-range temporal correlations, signal complexity, and fractal properties of EEG time series. Linear spectral features were calculated using the Welch periodogram method to estimate band power across standard EEG frequency bands. This approach provides robust spectral estimates by averaging modified periodograms across overlapping windows. More specific details were listed below.

**Largest Lyapunov Exponent (LLE)**

The largest Lyapunov exponent (LLE) quantifies the dynamical instability of a time series by measuring the exponential divergence of nearby trajectories in reconstructed phase space. Given a time series $S=[x(1),x(2),\ldots,x(N)]$, the phase space is reconstructed using time-delay embedding:

$$X(i)=[x(i),x(i+1),\ldots,x(i+m-1)]$$

where $m$ denotes the embedding dimension.

The divergence between two neighboring trajectories evolves as:

$$d_{j}(i)=d_{j}(0)e^{\lambda_{1}i\Delta t}$$

where $\lambda_{1}$represents the largest Lyapunov exponent. The LLE is estimated as the slope of the linear region of the logarithmic divergence curve:

$$\ln(d_{j}(i))=\ln(d_{j}(0))+\lambda_{1}i\Delta t$$

A positive LLE indicates chaotic dynamics and higher sensitivity to initial conditions. The LLE was computed to quantify the dynamical instability of EEG time series. Phase-space reconstruction was performed using embedding dimensions ranging from 5 to 20 with a time delay of 1 sample. The LLE was estimated from the slope of the linear region of the divergence curve using linear polynomial fitting across embedding dimensions 4-19.

**Detrended Fluctuation Analysis (DFA)**

DFA was used to evaluate long-range temporal correlations in EEG signals. The cumulative signal is first constructed:

$$y(k)=\sum_{i=1}^{k} [x(i)-\langle S\rangle]$$

where $\left\langle S \right\rangle$represents the mean of the time series.

The root mean square fluctuation for window size $n$is then calculated as:

$$F(n)=\sqrt{\frac{1}{N}\sum_{k=1}^{N} [y(k)-y_{n}(k)]^{2}}$$

The DFA scaling exponent $\alpha$is obtained from the relationship:

$$\alpha=\frac{\log(F(n))}{\log(n)}$$

This exponent characterizes the degree of long-range correlation in the signal. The DFA was used to quantify long-range temporal correlations in EEG signals. The box sizes ranged from 4 to 0.1 × N with a scaling factor of 1.2. Linear detrending (order = 1) was applied when estimating the fluctuation function.

**Approximate Entropy (ApEn)**

Approximate entropy quantifies the regularity and unpredictability of time-series data. For embedding dimension $m$ and tolerance $r$, vectors of length $m$are defined as:

$$x^{*}(i)=[x(i),x(i+1),...,x(i+m-1)]$$

The similarity probability $C_{i}^{m}(r)$is computed as:

$$C_{i}^{m}(r)=\frac{\sum_{j=1}^{N-m+1} \theta(r-\mid x^{*}(i)-x^{*}(j)\mid)}{N-m+1}$$

The approximate entropy is then defined as:

$$ApEn=\phi^{m}(r)-\phi^{m+1}(r)$$

where $\phi^{m}(r)$represents the logarithmic average of $C_{i}^{m}(r)$. Larger ApEn values indicate greater signal irregularity. The ApEn was computed to quantify signal regularity. The embedding dimension was set to $m$ = 2, and the tolerance threshold was defined as $r=0.2\times SD$, where SD denotes the standard deviation of the signal. The Chebyshev distance metric was used to evaluate vector similarity.

**Fractal Dimension**

Fractal dimension (FD) measures signal complexity and self-similarity. Two methods were used:

**Katz Fractal Dimension (KFD)**

KFD estimates signal complexity based on waveform geometry:

$$KFD=\frac{\ln(L/a)}{\ln(d/a)}$$

where $L$is the total signal length, $d$is the maximum distance between the first point and any other point, and $a$is the average step distance. The KFD was computed to characterize waveform complexity based on the geometric properties of the time series. This method does not require additional tunable parameters and was calculated directly from the time-series trajectory.

**Higuchi Fractal Dimension (HFD)**

HFD evaluates scale-invariant complexity by computing curve lengths at different time intervals $k$. The average curve length is defined as:

$$L(k)=\frac{1}{k}\sum_{m=1}^{k} L_{m}(k)$$

The fractal dimension is obtained from the slope of the relationship:

$$\log L(k)\sim\log(1/k)$$

Higher HFD values indicate greater signal complexity. The HFD was used to quantify scale-invariant signal complexity. The maximum interval parameter was set to $k_{max}=100$.

**Welch Periodogram**

Spectral features were computed using the Welch periodogram method to estimate the power spectral density (PSD):

$$P_{w}^{p}(f)=\frac{1}{P}\sum_{p=0}^{D-1} {\mid\frac{1}{UDT}\sum_{n=0}^{D-1} w[n]x^{p}[n]e^{-j2\pi fnT}\mid}^{2}$$

where $w[n]$represents the window function and $U$denotes the window energy:

$$U=T\sum_{n=0}^{D-1} w^{2}[n]$$

This method reduces spectral variance by averaging multiple windowed periodograms. Spectral power was subsequently extracted for standard EEG frequency bands. In the present study, the EEG signal was segmented using a Hamming window with 50% overlap to reduce spectral variance and improve estimation stability. Spectral features were computed using the Welch periodogram method to estimate the PSD. A Hanning window was applied, and the segment length (nperseg) was determined as $(1/f_{low})\times2\times250$Hz based on the sampling rate. Spectral power within each frequency band was subsequently calculated using Simpson’s rule integration.

**Machine learning methods**

SVM is a supervised ML algorithm for linear and nonlinear binary classification and could separate the data points in different classes by the hyperplanes with the largest optimal margin distance between each two classes [2]. The present study used Gaussian SVM with a radial basis function (rbf) kernel [3]. Hyperparameters were optimized using grid search with 10-fold cross-validation on the training dataset. The regularization parameter C and kernel parameter γ were searched over logarithmic ranges. The optimal parameters selected by cross-validation were C = 10 and γ = 0.05 for the rbf kernel.

RF employs the bagging concept to combine multiple decision tree classifiers into a single powerful model [4]. Such a bootstrap resampling approach is able to create new training sample sets from the initial N training samples by selecting random k (k<N) sets of samples repeatedly. The training sample is used to produce k buffeting decision trees for the construction of random forest, and the testing sample is then classified using majority vote decision. The strengths of RF include high generalization ability and low model variance. RF classifiers were constructed using an ensemble of decision trees. Hyperparameters, including the number of trees (n_estimators) and maximum tree depth (max_depth), were optimized using grid search with cross-validation on the training dataset. The optimal model selected by cross-validation consisted of 100 trees with no restriction on maximum tree depth.

XGBoost is named after extreme gradient boosting and is a scalable and efficient adaptive ML algorithm for tree boosting [5]. Tree boosting uses a powerful ensemble learning algorithm that combines many weak classifiers into a single strong classifier to improve classification accuracy. XGBoost provides parallel tree boosting and distributed computing that could solve problems in a fast and accurate way [6]. XGBoost also provides optimized algorithms, including a novel tree learning algorithm for handling sparse data, a theoretically justified weighted quantile sketch procedure for handling instance weights in approximate tree learning [5]. For the XGBoost classifier, hyperparameters including the number of estimators, learning rate, and L1 regularization term were optimized using cross-validation on the training dataset. The optimal configuration selected by cross-validation consisted of 150 estimators, a learning rate of 0.42, and a regularization parameter α = 0.1. The maximum tree depth was not explicitly restricted.

CatBoost is named after categorical boosting and is also one kind of gradient-boosting decision tree ML algorithm. It is well suited for categorical and heterogeneous data [7]. CatBoost is good at hyperparameter tuning by combing many categorical characteristics into a single one. It provides unbiased boosting with categorical features. CatBoost produces random permutations of the training data and collects gradients by sampling random permutations to improve the robustness of the algorithm. The hyperparameters were optimized using randomized search with cross-validation. The optimal model used 150 boosting iterations, a learning rate of 0.03, tree depth of 2, and an L2 regularization parameter of 3.

References

[1] Hyvarinen A, Oja E. Independent component analysis: algorithms and applications. Neural Netw. 2000; 13: 411-430. <https://doi.org/10.1016/s0893-6080(00)00026-5>

[2] Cortes C, Vapnik V. Support-Vector Networks. Machine Learning. 1995; 20: 273-297.

[3] Kousarrizi MRN, Ghanbari AA, Technehlab M, Shoorehdeli MA, Gharaviri A. Feature Extraction and Classification of EEG Signals using Wavelet Transform, SVM and Artificial Neural Networks for Brain Computer Interfaces. International Joint Conference on Bioinformatics, Systems Biology and Intelligent Computing. 2009: 352-355.

[4] Breiman L. Random forests. Machine Learning. 2001; 45: 5-32.

[5] Chen T, Guestrin C. XGBoost: A Scalable Tree Boosting System. KDD'16: Proceedings of the 22nd ACM SIGKDD International Conference on Knowledge Discovery and Data Mining. 2016: 785-794.

[6] Sharma A, Verbeke W. Improving Diagnosis of Depression With XGBOOST Machine Learning Model and a Large Biomarkers Dutch Dataset (n = 11,081). Front Big Data. 2020; 3: 15. <https://doi.org/10.3389/fdata.2020.00015>

[7] Hancock JT, Khoshgoftaar TM. CatBoost for big data: an interdisciplinary review. J Big Data. 2020; 7: 94. <https://doi.org/10.1186/s40537-020-00369-8>

Supplementary Table 1. Demographic and clinical variables among depressed patients with different levels of suicidality

|  | Age  mean (SD) | Male/Female (N/N) | HDRS-17  mean (SD) | MSM  mean (SD) |
| --- | --- | --- | --- | --- |
| No suicidality | 43.7 (13.9) | 14/23 | 14.0 (6.8) | 6.7 (1.8) |
| Mild | 40.5 (14.7) | 22/26 | 19.4 (5.2) | 8.2 (1.8) |
| Moderate | 35.4 (14.1) | 32/58 | 23.0 (5.9) | 8.8 (1.9) |
| Severe | 35.3 (14.0) | 13/15 | 26.8 (6.2) | 9.4 (2.1) |
| Profound | 38.3 (15.2) | 4/2 | 27.8 (3.6) | 10.7 (1.9) |
| F or *x*^2^/p-value | 3.01^a^ /0.22 | 3.69^b^ /0.45 | 32.6^a^ /<0.001 | 10.7^a^ /<0.001 |

^a^ one-way ANOVA; ^b^ chi-square test


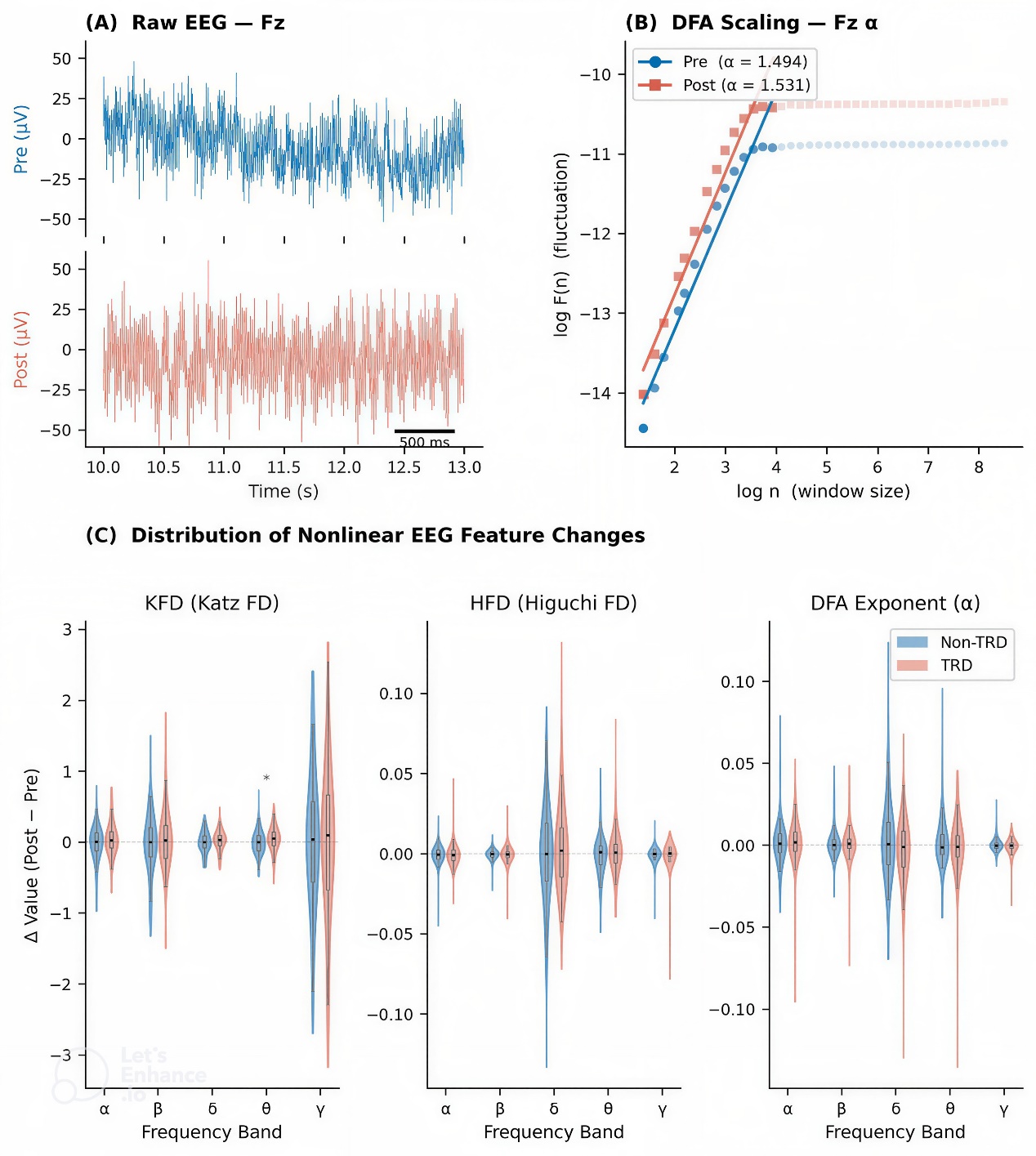


Supplementary Fig. 1. **Illustrative examples of nonlinear EEG feature extraction and distribution**. (A) A representative raw EEG time series obtained from the frontal electrode (Fz) before (Pre) and after (Post) the cognition-engaging task, demonstrating typical signal fluctuations used for nonlinear feature computation. (B) Example of dDetrended fFluctuation aAnalysis (DFA) scaling behavior in the alpha frequency band at the Fz electrode. The DFA exponent (α) is estimated from the slope of the linear relationship between log-transformed fluctuation magnitude 𝐹(𝑛) and window size 𝑛, reflecting long-range temporal correlations in the EEG signal. (C) Distribution of changes in nonlinear EEG complexity measures (Post-Pre) across frequency bands for Katz fFractal dDimension (KFD), Higuchi fFractal dDimension (HFD), and the DFA exponent. Violin plots illustrate the variability of feature changes across participants, with comparisons between non-treatment-resistant depression (non-TRD) and treatment-resistant depression (TRD) groups. * A minimal between-group difference (*p* < 0.05) was observed through exploratory univariate comparisons. However, the size of individual feature variations was often minor, implying that single EEG features may have limited discriminatory ability. These examples show how nonlinear EEG measures capture signal complexity and temporal dynamics, which may aid in the machine learning-based classification of clinical characteristics in major depressive disorder. EEG, electroencephalography; FD, Fractal Dimension.
